# Multi-Feature Nonlinear Optimization Motion Estimation Based on RGB-D and Inertial Fusion

**DOI:** 10.3390/s20174666

**Published:** 2020-08-19

**Authors:** Xiongwei Zhao, Cunxiao Miao, He Zhang

**Affiliations:** School of Mechanical Engineering, University of Science and Technology Beijing, Beijing 100083, China; g20188592@xs.ustb.edu.cn (X.Z.); b20180257@xs.ustb.edu.cn (H.Z.)

**Keywords:** nonlinear optimization, multiple features, motion estimation, RGB-D visual-inertial odometry, sliding windows

## Abstract

To achieve a high precision estimation of indoor robot motion, a tightly coupled RGB-D visual-inertial SLAM system is proposed herein based on multiple features. Most of the traditional visual SLAM methods only rely on points for feature matching and they often underperform in low textured scenes. Besides point features, line segments can also provide geometrical structure information of the environment. This paper utilized both points and lines in low-textured scenes to increase the robustness of RGB-D SLAM system. In addition, we implemented a fast initialization process based on the RGB-D camera to improve the real-time performance of the proposed system and designed a new backend nonlinear optimization framework. By minimizing the cost function formed by the pre-integrated IMU residuals and re-projection errors of points and lines in sliding windows, the state vector is optimized. The experiments evaluated on public datasets show that our system achieves higher accuracy and robustness on trajectories and in pose estimation compared with several state-of-the-art visual SLAM systems.

## 1. Introduction

In recent years, simultaneous localization and mapping (SLAM) [1] has become an attractive research topic in many self-localization robotic areas, particularly with the development of mobile robots. The SLAM technology can estimate a robot’s own motion and attitude using sensors without any prior information of the environment. Consequently, it is widely used for autonomous cars, virtual reality and augmented reality, unmanned air vehicles, and indoor robots. For the localization of indoor robots, high-precision map and global positioning system [2] technologies are not typically available indoors, and low-precision inertial sensors can barely meet the requirements of accuracy and applicability. A visual-inertial odometry (VIO) that combines the information of cameras and IMU has evidently improved the accuracy and robustness for the localization of indoor robots. Cameras can capture rich feature information by processing images, and IMU can obtain accurate motion estimation of robots in a short timeframe, which reduces the impact of dynamic objects on cameras. Further, cameras can provide effective corrections for inertial drifts. To a certain extent, these two sensors can complement each other, and the VIO system has been popularized for use in various potential applications.

Based on whether image feature information is incorporated into the system state vector, a typical VIO system can be divided into a loosely coupled method and a tightly coupled method [3,4]. A loosely coupled method implies that the camera and IMU generate each their own motion estimation, and then the VIO system fuses the position and attitude of these two estimation results. Meanwhile, a tightly coupled method establishes motion and observation equations by combining raw measurements from the camera and IMU. Compared with the former, the latter generally has better accuracy and robustness. In this study, the proposed system is a tightly coupled VIO system. For the system state estimation, a filter-based approach and an optimization-based approach are both used [5,6]. Filtering approaches, based on the extended Kalman filter (EKF), use IMU measurements for system state estimation, while visual measurements are designed to update the latest state. Mourikis et al. [7] presented a multi-state constraint Kalman filter based on a filtering-based approach, whose backend predicted and updated the state vectors with EKF in sliding windows. However, the drawback of this system is that not all current camera information is used in the filter and that this system processes the measurements of sensors only once per updating step. Optimization-based approaches obtain an optimal estimate by minimizing a joint nonlinear cost function with all IMU and visual residuals. Thus, optimization-based approaches usually achieve a higher accuracy than filtering-based methods. Stefan et al. [8] proposed an open keyframe visual-inertial SLAM (OKVIS) based on a nonlinear optimization framework, which uses IMU pre-integration [9] to avoid repeated IMU integration and the first-in-last-out sliding window method for bounding computation to handle all measurements. Qin et al. [10] also studied a nonlinear-optimization framework, VINS-MONO, which selectively marginalizes visual landmarks and achieves an accurate VIO system for monocular system. Their work enables optimization-based methods to achieve better real-time performance. However, the above VIO systems are based on monocular and stereo cameras. Monocular cameras cannot directly obtain depth information of visual features and require a complex initialization process to recover the scale of visual features, leading to system-scale uncertainty and increases in the computational complexity. Stereo also requires high computing cost to generate the corresponding depth information. Compared with these methods, RGB-D cameras can obtain both color images and aligned depth information, which can simplify the triangulation of point features and achieve a faster initialization process. However, few works have integrated IMU with RGB-D camera measurements for a VIO system up until now. Yang et al. [11] presented an RGB-D-inertial odometry via keyframe-based nonlinear optimization. A non-iterative odometry was proposed in [12], which used a kernel cross-correlator (KCC) to match point clouds and combine IMU measurements to generate a dense indoor map. Although the above mentioned RGB-D-inertial systems, which rely only on point features, have improved the localization accuracy in most scenes, point detection and matching in textureless environments exhibit a poor performance. In contrast, line features provide more structural information of these scenes than points. There are some monocular SLAM systems that combine point and line features. He et al. [13] proposed the PL-VIO system, which is based on VINS-MONO. Although the PL-VIO has achieved good accuracy on public datasets, it needs a complex initialization process and consumes a lot of computing resources because of system-scale uncertainty of the monocular system. For an RGB-D SLAM system, several works combined point and line features to estimate camera motion [14,15]. Lu et al. [16] proposed the LineSLAM, which detected line segments from depth images of RGB-D cameras and implemented an RGB-D visual odometer by combining points and lines. This system is currently the latest open source RGB-D system based on points and lines. However, without the combination of other sensor data, the LineSLAM will not work when the visual information is lost. Plücker coordinates were first introduced to represent 3D lines in [17], which also used orthonormal representation to handle the nonlinear optimization of line features and avoid over-parameterizations. For an RGB-D-inertial SLAM system, no work that combines point and line features has been conducted yet. Based on the previous discussion, a tightly coupled RGB-D visual-inertial odometry based on multi-features is presented in this paper. The key contributions are as follows:

- To our best knowledge, the proposed system is the first tightly coupled optimization-based RGB-D-inertial system based on points and lines.
- At the beginning of the system, 3D point features can be directly obtained from depth images produced by the RGB-D camera. To correct IMU bias at the beginning, we implemented a fast initialization aligning vision-only measurements with the values of IMU pre-integration.
- This paper achieves an RGB-D-inertial nonlinear optimization framework with constraints of both the IMU kinematic model and the reprojection of points and lines in sliding windows. An orthonormal representation is employed to parameterize line segments and analytically calculate the corresponding Jacobians.
- A series of experiments has been designed to compare the performance of our system with the other four open-source visual SLAM systems: PLVIO [13], OKVIS [8], VINS-MONO [10] and LineSLAM [16]. The results validate the accuracy and robustness of the system proposed herein.

## 2. Mathematical Formulation

### 2.1. Notations and Definitions

**Figure 1:**
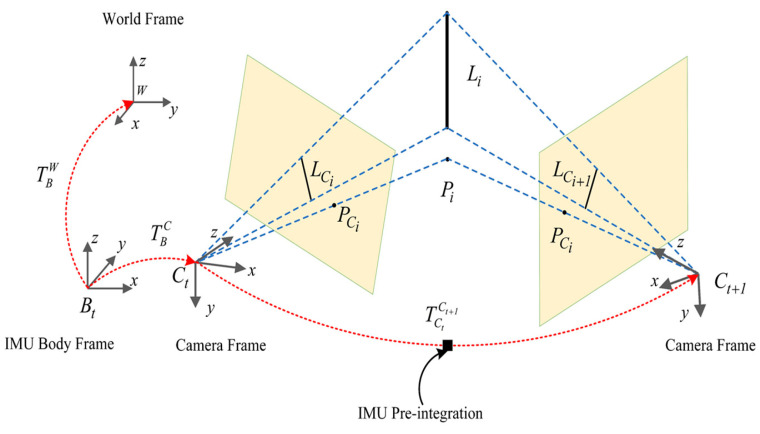
Symbol definitions of the system.

The notations used and details throughout this work are as shown in Figure 1. ${\left(\cdot \right)}_{C}$ is the camera frame; ${\left(\cdot \right)}_{B}$ denotes the body frame, which is the same as the IMU frame; ${\left(\cdot \right)}_{W}$ is the world frame, and ${\left[\cdot \right]}_{\times }$ is the skew-symmetric matrix of the vector. We denote $T_{B}^{C}\in SE\left(3\right)$ as the homogeneous transformation matrix from the body frame to the camera frame, $T_{B}^{C}=\left(R_{B}^{C},t_{B}^{C}\right)$. $L_{i}$ and $P_{i}$ are the *i*th point landmark and the line landmark, respectively.

### 2.2. IMU Pre-Integration

Inertia devices usually consist of an accelerometer and a gyroscope, which measure the three-axis acceleration a and the three-axis angular velocity w. Owing to the influences of internal mechanical accuracy and temperature factors, white noise and bias occur in IMU measurements. The measurement models of IMU are expressed as follows:

(1) $${\hat{w}}_{B}=w_{B}+b^{w}+\eta^{w}$$

(2) $${\hat{a}}_{B}=R_{W}^{B}\left(a_{W}-g_{W}\right)+b^{a}+\eta^{a}$$

where $b^{w}$ and $b^{a}$ denote a gyroscope and a accelerometer bias, respectively, which are usually modeled as a random walk process. Further, $\eta^{w}$ and $\eta^{a}$ are the corresponding Gaussian white noise process, ${\hat{w}}_{B}$ and ${\hat{a}}_{B}$ are the raw measurements by IMU, and $a_{W}$ and $g_{W}={\left[0,0,g\right]}^{T}$ represent the true acceleration and gravity, respectively, in the world frame. Given a system state, namely, position $p_{B}^{W}$, velocity $v_{B}^{W}$, and quaternions $q_{B}^{W}$, we can obtain all body states in discrete form between consecutive frames $B_{k}$ and $B_{k+1}$ in the world frame according to IMU’s kinematic model.

(3) $$R_{W}^{B_{k}}p_{B_{k+1}}^{W}=R_{W}^{B_{k}}\left(p_{B_{k}}^{W}+v_{B_{k}}^{W}\Delta t_{}-\frac{1}{2}g_{W}\Delta t_{}^{2}\right)+\alpha_{B_{k+1}}^{B_{k}}$$

(4) $$R_{W}^{B_{k}}v_{B_{k+1}}^{W}=R_{W}^{B_{k}}\left(v_{B_{k}}^{W}-g_{W}\Delta t_{}^{}\right)+\beta_{B_{k+1}}^{B_{k}}$$

(5) $$q_{W}^{B_{k}}⊗q_{B_{k+1}}^{W}=\gamma_{B_{k+1}}^{B_{k}}$$

where, $\left[\begin{array}{ccc} \alpha_{B_{k+1}}^{B_{i}} & \beta_{B_{k+1}}^{B_{i}} & \gamma_{B_{k+1}}^{B_{i}} \end{array}\right]$ are called IMU pre-integration [9] and can be directly calculated without knowledge of the system state, indicating that when a body state is changed, re-propagation of IMU is not necessary. Time t=i is between time interval $\left[t_{k},t_{k+1}\right]$. To save computational resources, the biases $b_{}^{a},b_{}^{w}$ are set to constant during the pre-integration between consecutive frames.

### 2.3. Representation of 3D Line Features

Traditional line segmentation detection usually uses the Canny algorithm [18] to obtain edge information in an image and then uses the Hough transform to extract line features. However, this method is time consuming, and many false detections may occur when edge features are dense. Line segment detector (LSD) [19] is a line detection and segmentation algorithm, which can obtain sub-pixel accuracy detection results in linear time. The LSD algorithm is used to detect and match two endpoints of line features in this study, whereas the merged line features are represented by LBD line descriptors [20]. Based on LSD line detection, we introduce the geometric characteristics of the line to match line pairs effectively. In this study, we set the following conditions for line matching: (1) the angular difference of matched line pairs is smaller than a given threshold $\theta =30^{\circ }$ and (2) the distance between two LBD descriptors is less than the value δ=60 pixel.

**Figure 2:**
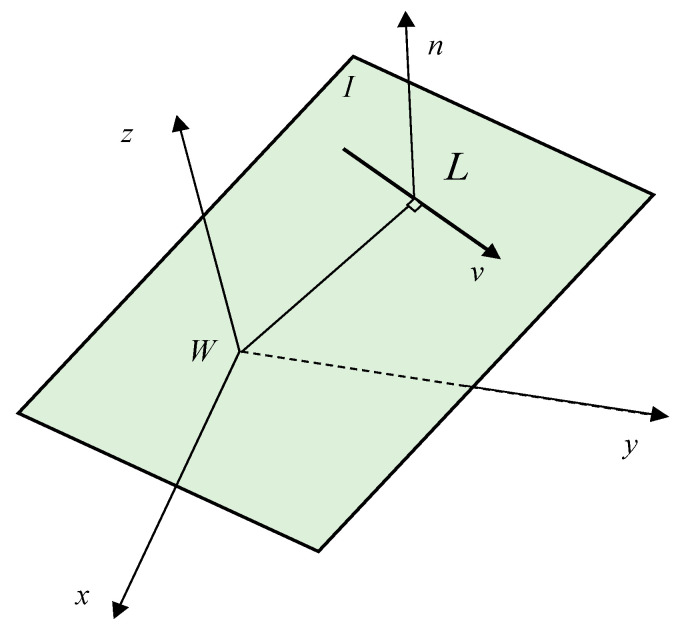
Spatial line and Plücker coordinates.

As a 3D line segment L can be represented by its two endpoints, Plücker coordinates are introduced for a more compact representation. We assume that the homogeneous coordinates of two endpoints are $X_{1}={\left(x_{1},y_{1},z_{1},w_{1}\right)}^{T}$ and $X_{2}={\left(x_{2},y_{2},z_{2},w_{2}\right)}^{T}$, while their inhomogeneous coordinates are denoted by ${\tilde{X}}_{1},{\tilde{X}}_{2}$. The Plücker coordinates of a line segment can be constructed as follows:

(6) $$L=\left[\begin{array}{c} {\tilde{X}}_{1}\times {\tilde{X}}_{2}\\ w_{2}{\tilde{X}}_{1}-w_{1}{\tilde{X}}_{2} \end{array}\right]=\left[\begin{array}{c} n\\ v \end{array}\right]\in P^{5}\subset R^{6}$$

where $v\in R^{3}$ is the direction vector of the line feature, and $n\in R^{3}$ is the normal vector of plane I as determined by line segments and world coordinate origin, which are shown in Figure 2. Their relationship is $n^{T}v=0$.

For the line geometric characteristics, we can obtain the transformation of the Plücker coordinates from the world frame to the camera frame from Equation (7).

(7) $$L_{C}=\left[\begin{array}{c} n_{C}\\ v_{C} \end{array}\right]=T_{W}^{C}L_{W}=\left[\begin{array}{cc} R_{W}^{C} & {\left[t_{W}^{C}\right]}_{\times }R_{W}^{C}\\ 0 & R_{W}^{C} \end{array}\right]L_{W}$$

where ${\left[\cdot \right]}_{\times }$ is the skew-symmetric matrix of the vector. According to a pin-hole model camera, a spatial line represented by Plücker coordinates can be projected to the image plane as in [21].

(8) $$l_{C}=\left[\begin{array}{c} l_{1}\\ l_{2}\\ l_{3} \end{array}\right]=KL_{C}=\left[\begin{array}{ccc} f_{v} & 0 & 0\\ 0 & f_{u} & 0\\ -f_{v}c_{u} & -f_{u}c_{v} & f_{u}f_{v} \end{array}\right]L_{C}$$

where K denotes the projection matrix of the camera. As a straight line only has four degrees of freedom, Plücker coordinates, which consist of six parameters, are over-parameterized. At the graph optimization, the extra degrees of freedom will increase the computational cost, resulting in an instability in the system. Therefore, an orthonormal coordinate [22] consisting of four parameters is proposed to optimize the line features. The orthonormal representation (U,W)∈SO(3)SO(2) is more suitable than Plücker coordinates for optimization and they can easily be converted into each other. The orthonormal representation of line features is denoted as follows:

(9) $$l_{o}=\left[\begin{array}{cc} n & v \end{array}\right]=\left[\begin{array}{ccc} \frac{n}{\left\|n\right\|} & \frac{n}{\left\|v\right\|} & \frac{n\times v}{\left\|n\times v\right\|} \end{array}\right]\left[\begin{array}{cc} \left\|n\right\| & 0\\ 0 & \left\|v\right\|\\ 0 & 0 \end{array}\right]=U\left[\begin{array}{cc} w_{1} & 0\\ 0 & w_{2}\\ 0 & 0 \end{array}\right]$$

where the orthogonal matrix U represents the rotation between the line coordinates and the camera frame. Matrix W represents the distance information from the camera coordinate origin to the 3D line. The orthonormal representation (U,W) consists of:

(10) $$U=U\left(\varphi \right)=\left[\begin{array}{ccc} \frac{n}{\left\|n\right\|} & \frac{v}{\left\|v\right\|} & \frac{n\times v}{\left\|n\times v\right\|} \end{array}\right]$$

(11) $$W=\left[\begin{array}{cc} w_{1} & -w_{2}\\ w_{2} & w_{1} \end{array}\right]=\left[\begin{array}{cc} cos\left(\phi \right) & -sin\left(\phi \right)\\ sin\left(\phi \right) & cos\left(\phi \right) \end{array}\right]=\frac{1}{\sqrt{\left({\left\|n\right\|}^{2}+{\left\|v\right\|}^{2}\right)}}\left[\begin{array}{cc} \left\|n\right\| & -\left\|v\right\|\\ \left\|v\right\| & \left\|n\right\| \end{array}\right]$$

We can update the orthonormal representation with a minimum of four parameters $\theta ={\left[\begin{array}{cc} \varphi & \phi \end{array}\right]}^{T}$ in the process of optimization. Matrix U is updated by φ, which are the rotation angles of the three axes in the camera frame. Matrix W is updated by ϕ, which is the translation of a 3D line in the camera frame.

## 3. Overall Structure of the VIO System

**Figure 3:**
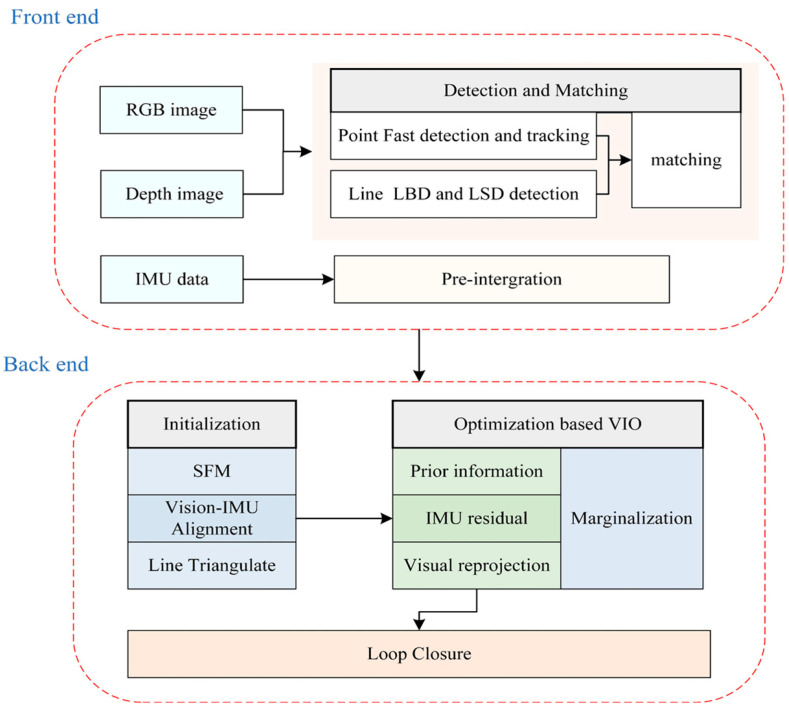
Overview of system structure.

The proposed system structure is shown in Figure 3. It mainly includes two parts: the frontend and the backend. At the beginning of the system, the data from IMU and the RGB-D camera are received and processed at the frontend module, where the transformation between two consecutive frames is obtained through feature detection and matching. Further, the corresponding system initial pose of IMU pre-integration is provided. Histogram equalization will be conducted on the original RGB image [23], which strengthens the adaptability of images to scenes with light changes for better extraction of features. When a new frame is received, the point features are tracked between the previous frame and the new frame by the KLT sparse optical flow algorithm [24]. Then, the RANSAC framework with a fundamental matrix was used to remove outliers by establishing a parametric model [25]. We used the LSD algorithm to detect line features whose threshold was set to assure that an adequate number of features can be detected. A FAST detector [26] was used to maintain a adequate number of points features in each image. The depth image and RGB image were aligned by hardware, and depth filtering was performed to reduce corresponding errors. We can determine the aligned depth information of each feature from the depth image according to the projection model of the pinhole camera. Meanwhile, the strategy for selecting the keyframe depends on whether the average parallax of the point feature between the current frame and the latest frame is beyond a specified threshold.

In the backend, first, with a set of corresponding 3D points features, the iterative closest point (ICP) algorithm was used to recover the initial pose in the process of structure from motion (SFM). We maintained a certain number of frames in a sliding window to reduce the computational complexity. The line features were triangulated to set up re-projection residuals. After obtaining the pose of all frames and 3D landmarks form vision-only SFM in the sliding windows, we aligned the poses with the measurements of the IMU pre-integration and the visual measurements to obtain a optimal initial state. We built a nonlinear optimization framework to minimize the residuals of the visual re-projection, IMU measurements and prior information of marginalization. To prevent the number of poses and features from increasing excessively, we used a marginalization strategy to reduce the computational complexity of our system. Finally, we integrated the Loop Closure to reduce cumulative errors in pose estimation and improve the accuracy of the system.

## 4. Nonlinear Optimization Framework

**Figure 4:**
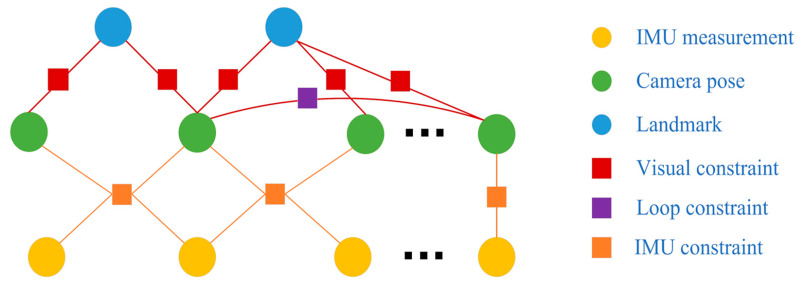
Factor graphs of our system.

In this section, the entire backend structure is detailed. To reduce the computational complexity of the backend process, we used a nonlinear optimization method based on sliding windows, in which only a certain number of frames were maintained. A system marginalization strategy was also applied to exclude the oldest frame in a window and add prior information into the next local window optimization. We derived the state constraints of the system through a factor graph, identified the state vector to be optimized and established a nonlinear optimization equation. Meanwhile, to ensure the global consistency of the system, a global loop closure module was incorporated. As shown in Figure 4, circular nodes were the states that needed to be optimized, which included the camera pose and landmarks. Rectangular nodes were the system constraints of the visual measurements, IMU measurements and loop information.

### 4.1. System State

The full state variables that needed to be optimized in a sliding window are defined as follows:

(12) $$\eta ={\left[x_{n},x_{n+1},\ldots ,x_{n+N},l_{m},l_{m+1},\ldots ,l_{m+M}\right]}^{T}$$

(13) $$x_{i}={\left[p_{B_{i}}^{W},q_{B_{i}}^{W},\nu_{B_{i}}^{W},b_{a}^{},b_{g}^{}\right]}^{T},i\in \left[n,n+N\right]$$

where η is the state vector that contains point and line states. *N* and *M* are the numbers of keyframes and line landmarks observed, respectively, by all keyframes in the sliding window. $l_{m}$ denotes the orthonormal representation of the *m*-th line feature in the world frame. For the *i*-th frame image, $x_{i}$ is the position, velocity, orientation in the world frame and the bias term for the accelerometer and gyroscope, respectively, of the corresponding IMU state.

First, the first frame was set as a keyframe, and then the next keyframe was selected according to our strategy. We sought a bundle adjustment formulation to jointly minimize the residuals of all measurements in the Mahalanobis distance. There were mainly four parts of residuals in our system. The cost function is defined as

(14) $$\underset{\eta }{min}\left\{\rho \left({\left\|r_{M}-J_{M}\eta \right\|}_{\sum M}^{2}\right)+\sum_{i\in B}\rho \left({\left\|r_{B}\left(z_{B_{i+1}}^{B_{i}},\eta \right)\right\|}_{P_{B_{k+1}}^{B_{k}}}^{2}\right)+\sum_{i\in C,j\in P}\rho \left({\left\|r_{p}\left(z_{C_{i+1}}^{C_{i}},\eta \right)\right\|}_{\sum p_{j}}^{2}\right)+\sum_{\left(i,j\right)\in L}\rho \left({\left\|r_{l}\left(z_{L_{i}}^{C_{i}},\eta \right)\right\|}_{\sum {}_{L_{i}}^{c_{i}}}^{2}\right)\right\}$$

where $r_{M}$ and $J_{M}$ are the prior information from marginalization, which contain the constraints of IMU measurements, point and line features removed from the sliding windows [27]. ρ is the Huber robust function used to decrease the influence of outliers on the whole state estimation. $r_{B}$ is the residual of the IMU pre-integration between two consecutive frames. $r_{P}$ and $r_{l}$, respectively, represent the residuals of point measurements and line re-projections. *B* is the set of all IMU measurements. *P* and *L* are separately the sets of landmarks and spatial lines, both of which are observed many times in the windows, *C* is the set of all keyframes.

### 4.2. System Initialization

Visual-inertial odometry is a highly nonlinear system, and there exists IMU bias during the operation of the system, hence, system initialization is very important for correcting initial IMU bias and achieving a high-precision position. In our initialization strategy, we used a loose-coupled method to fuse data from two sensors, with the RGB-D camera and IMU running separately for a short time to calculate the corresponding poses. Then, we aligned the poses from visual structure from motion (SFM) and the IMU pre-integration to correct the initial error of the IMU. Our process of initialization was divided into the following modules.

#### 4.2.1. Structure from Motion (SFM)

The monocular camera could not determine an absolute scale of feature in 3D space, while the RGB-D camera could quickly obtain more accurate depth information. With the depth information from RBG-D camera, the 3D landmarks of the matched point features between the two frame could be obtained. Firstly, a visual SFM was taken to find the frame which had enough matching feature points with the current frame in the sliding windows. The selected keyframes were used as a reference frame for pose estimation in turn, which could recover the rotation matrix and translation vector between two consecutive frames. According to the pinhole camera model, we could obtain two sets of matched 3D points between the *i*-th frame and the *j*-th frame, which are denoted as:

(15) $$P=\left\{p_{i}|p_{i}\in R^{3},i=1,2,\cdots ,n\right\}$$

(16) $$Q=\left\{q_{j}|q_{j}\in R^{3},i=1,2,\cdots ,n\right\}$$

The corresponding transformation is described in Equation (17), and we used an ICP algorithm to estimate the state of 3D–3D matching. We could turn this into a least square problem with Equation (18). In addition, we solved Equation (18) using singular value decomposition (SVD) to obtain the pose of the visual-only SFM.

(17) $$p_{i}=R_{C_{j}}^{C_{i}}q_{j}+t_{C_{j}}^{C_{i}}$$

(18) $$\underset{T_{C_{i}C_{j}}}{\arg\min}=\frac{1}{2}{\sum \left\|p_{i}-\left(R_{C_{j}}^{C_{i}}q_{j}+t_{C_{j}}^{C_{i}}\right)\right\|}_{2}^{2}$$

#### 4.2.2. Visual-IMU Alignment

During initialization, for all consecutive frames, the rotation matrix measured by the camera should have been theoretically the same as that measured by the IMU. However, in reality, they were not exactly the same because of an existing gyroscope bias. Hence, we built the following cost function to minimize the relative rotation error of them:

(19) $$\underset{\delta b_{w}}{\min}{\sum_{i\in F}\left\|q_{B_{i}}^{B_{i+1}}⊗\gamma_{B_{i+1}}^{B_{i}}\right\|}^{2}$$

where $q_{B_{i}}^{B_{i+1}}$ represents the rotation matrix of consecutive frames $B_{i}$ to $B_{i+1}$ measured by the visual-only SFM, and $\gamma_{B_{i+1}}^{B_{i}}$ is the rotation measurement measured by IMU pre-integration.

### 4.3. IMU Measurement Residual

In our system, the IMU measurement residual was the norm of the difference between the observed and estimated values of IMU in the Mahalanobis distance. Considering adjacent frames *i* and *i* + 1, we obtained the predicted motion of frame *i* + 1 by using related IMU data of frame *i* to create the pre-integrated term; the IMU pre-integrated residual is described as:

(20) $$r_{B}\left(z_{B_{i+1}}^{B_{i}},\eta \right)={\left[\begin{array}{ccccc} \delta p & \delta v & \delta q & \delta b_{a} & \delta b_{g} \end{array}\right]}^{T}=\left[\begin{array}{c} R_{W}^{B_{i}}\left(p_{B_{i+1}}^{W}-p_{B_{i}}^{W}+\frac{1}{2}g^{W}\Delta t^{2}-v_{B_{i}}^{W}\Delta t_{i}\right)-{\hat{\alpha }}_{B_{i+1}}^{B_{i}}\\ R_{W}^{B_{i}}\left(v_{B_{i+1}}^{W}+g^{W}\Delta t_{i}-v_{B_{i}}^{W}\right)-{\hat{\beta }}_{B_{i+1}}^{B_{i}}\\ 2{\left[q_{B_{i}}^{W^{-1}}⊗q_{B_{i+1}}^{W}⊗{\left({\hat{\gamma }}_{B_{i+1}}^{B_{i}}\right)}^{-1}\right]}_{xyz}\\ b_{a}{}_{B_{i+1}}-b_{a}{}_{B_{i}}\\ b_{g}{}_{B_{i+1}}-b_{g}{}_{B_{i}} \end{array}\right]$$

where $\left[\begin{array}{ccc} {\hat{\alpha }}_{B_{i+1}}^{B_{i}} & {\hat{\beta }}_{B_{i+1}}^{B_{i}} & \gamma_{B_{i+1}}^{B_{i}} \end{array}\right]$ represents the IMU pre-integrated term between two consecutive image frames. ${\left[\cdot \right]}_{xyz}$ denotes the error of the three-dimensional rotation vector.

### 4.4. Visual Reprojection Residual

For point features, *j*-th feature points in the sliding window were observed by the two matched frames *I* and *i* + 1. According to the RGB-D camera model, we could directly obtain the corresponding 3D coordinates $P_{C_{i}}^{j}$ and $P_{C_{i+1}}^{j}$, ${\hat{P}}_{C_{i}}^{j}$ was the projection point of $P_{C_{i}}^{j}$ from frame $C_{i}$ to $C_{i+1}$. The residual of the *j*-th feature points is defined as:

(21) $$r_{p}\left(z_{C_{i+1}}^{C_{i}},x\right)=\left\|{\hat{P}}_{C_{i}}^{j}-P_{Ci+1}^{j}\right\|=\left[\begin{array}{c} {\hat{X}}_{i+1}-X_{i+1}\\ {\hat{Y}}_{i+1}-Y_{i+1}\\ {\hat{Z}}_{i+1}-Z_{i+1} \end{array}\right]$$

where:

(22) $$\left[\begin{array}{c} {\hat{P}}_{C_{i+1}}^{j}\\ 1 \end{array}\right]=T_{B}^{C}T_{W}^{B_{i+1}}T_{B_{i}}^{W}T_{C}^{B}\left[\begin{array}{c} P_{C_{i}}^{j}\\ 1 \end{array}\right]=\left[\begin{array}{c} R_{B}^{C}\left(R_{W}^{B_{i+1}}\left(R_{W}^{B_{i}{}^{T}}\left(R_{B}^{C^{T}}P_{C_{i}}^{j}+P_{B}^{C^{T}}\right)+P_{B_{i}}^{W}\right)+P_{W}^{B_{i+1}}\right)+P_{B}^{C}\\ 1 \end{array}\right]$$

To minimize the point measurement residuals, the Levenberg–Marquard algorithm was applied to solve the cost function, which can be denoted as:

(23) $$\underset{x}{\min}\sum_{i\in C,i\in P}{\left\|r_{p}\left(z_{C_{i+1}}^{C_{i}},x+\Delta x\right)\right\|}_{\sum p_{j}}^{2}=\underset{x}{\min}{\left\|r_{p}\left(z_{C_{i+1}}^{C_{i}},x\right)+J_{C_{i+1}}^{C_{i}}\Delta x\right\|}_{\sum p_{j}}^{2}$$

where $J_{C_{i+1}}^{C_{i}}$ is the Jacobian matrix of residual $r_{p}$ with respect to x. Therefore, we must derive the rotation and translation of frame $C_{i}$, $C_{i+1}$ for $J_{C_{i+1}}^{C_{i}}$:

(24) $$J_{C_{i+1}}^{C_{i}}=\left[\begin{array}{cc} \frac{\partial r_{p}}{\partial x_{i}} & \frac{\partial r_{p}}{\partial x_{i+1}} \end{array}\right]$$

With

(25) $$\frac{\partial r_{p}}{\partial x_{i}}={\left[\begin{array}{ccccc} R_{B}^{C} & -R_{B}^{C}R_{W}^{B_{i+1}}R_{B_{i}}^{W}{\left[R_{C}^{B}P_{C_{i}}^{j}+p_{C}^{B}\right]}_{\times } & 0 & 0 & 0 \end{array}\right]}_{3\times 15}$$

(26) $$\frac{\partial r_{p}}{\partial x_{i+1}}={\left[\begin{array}{ccccc} -R_{B}^{C}R_{W}^{B_{j}} & R_{B}^{C}{\left[R_{W}^{B_{i+1}}R_{B_{i}}^{W}\left(R_{C}^{B}P_{C_{i}}^{j}+P_{C}^{B}\right)\right]}_{\times } & 0 & 0 & 0 \end{array}\right]}_{3\times 15}$$

After we obtained all Jacobians for the system state, the Levenberg–Marquard algorithm in the Ceres solver library [28] was used to find the optimal state x.

**Figure 5:**
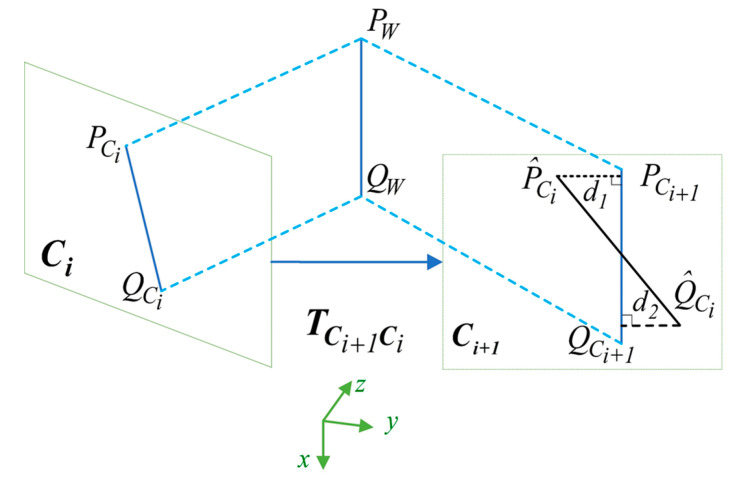
Interpretation of the line feature reprojection error.

For the line features, a suitable residual model of line segments must also be developed. According to Equation (7), the spatial 3D line $l_{W}$ in the world frame can be converted to $l_{C}$ in the camera frame. $l_{W}$ and $l_{C}$ are represented in Plücker coordinates. In this study, we defined the difference between the position of a line segment detected in the image plane as the re-projection residual of lines. As shown in Figure 5, $P_{W}Q_{W}$ is the spatial line feature observed in the two keyframes $C_{i}$ and $C_{i+1}$ in the world frame. $P_{C_{i}}Q_{C_{i}}$ and $P_{C_{i+1}}Q_{C_{i+1}}$ are, respectively, the observation model of line $P_{W}Q_{W}$ in frame $C_{i}$ and frame $C_{i+1}$. ${\hat{P}}_{C_{i}}{\hat{Q}}_{C_{i}}$ detected in frame $C_{i+1}$ is considered to be matched with $P_{C_{i}}Q_{C_{i}}$, which usually does not coincide with $P_{C_{i+1}}Q_{C_{i+1}}$. Thus, the line re-projection residual denotes a sum of point-to-line distances from two endpoints of ${\hat{P}}_{C_{i}}{\hat{Q}}_{C_{i}}$ to line $P_{C_{i+1}}Q_{C_{i+1}}$:

(27) $$r_{l}\left(z_{L_{i}}^{C_{i}},\eta \right)={\left[\begin{array}{cc} \frac{{\hat{P}}_{C_{i+1}}^{T}l_{C_{i+1}}}{\sqrt{l_{1}^{2}+l_{2}^{2}}} & \frac{{\hat{Q}}_{C_{i+1}}^{T}l_{C_{i+1}}}{\sqrt{l_{1}^{2}+l_{2}^{2}}} \end{array}\right]}^{T}$$

To optimize the cost function, we also used the Levenberg–Marquard algorithm, the corresponding Jacobian matrix with respect to the increment of the system state and the four dimensional vector θ, which updates the orthonormal representation:

(28) $$\frac{\partial r_{l}}{\partial x}=\frac{\partial r_{l}}{\partial l_{C_{i+1}}}\frac{\partial l_{C_{i+1}}}{\partial L_{C}}\left[\begin{array}{cc} \frac{\partial L_{C}}{\partial x} & \frac{\partial L_{C}}{\partial \theta } \end{array}\right]$$

with:

(29) $$\frac{\partial r_{l}}{\partial l_{C}}=\left[\begin{array}{ccc} \frac{u_{1}l_{2}^{2}-l_{1}l_{2}v_{1}-l_{1}l_{3}}{{\left(l_{1}^{2}+l_{2}^{2}\right)}^{\frac{3}{2}}} & \frac{v_{1}l_{2}^{2}-l_{1}l_{2}u_{1}-l_{2}l_{3}}{{\left(l_{1}^{2}+l_{2}^{2}\right)}^{\frac{3}{2}}} & \frac{1}{\sqrt{l_{1}^{2}+l_{2}^{2}}}\\ \frac{u_{2}l_{2}^{2}-l_{1}l_{2}v_{2}-l_{1}l_{3}}{{\left(l_{1}^{2}+l_{2}^{2}\right)}^{\frac{3}{2}}} & \frac{v_{2}l_{2}^{2}-l_{1}l_{2}v_{2}-l_{2}l_{3}}{{\left(l_{1}^{2}+l_{2}^{2}\right)}^{\frac{3}{2}}} & \frac{1}{\sqrt{l_{1}^{2}+l_{2}^{2}}} \end{array}\right]$$

(30) $$\frac{\partial l_{C_{i+1}}}{\partial L_{C}}={\left[\begin{array}{cc} K & 0 \end{array}\right]}_{3\times 6}$$

(31) $$\frac{\partial L_{C}}{\partial \theta }=\frac{\partial L_{C}}{\partial L_{W}}\frac{\partial L_{W}}{\partial \theta }=T_{W}^{C}{\left[\begin{array}{cc} -{\left[w_{1}u_{1}\right]}_{\times } & -w_{2}u_{1}\\ -{\left[w_{2}u_{2}\right]}_{\times } & w_{1}u_{2} \end{array}\right]}_{6\times 4}$$

### 4.5. Marginalization

**Figure 6:**
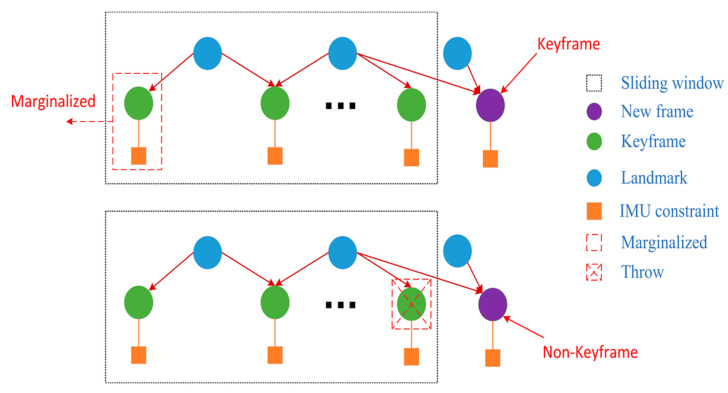
Marginalization strategy of our system.

Owing to the limited computational resources of most indoor robots, it was difficult to optimize all camera poses and landmarks. Therefore, the IMU states, feature points and feature lines were selectively marginalized from the sliding windows. Thus, an optimal computational complexity was maintained, within an acceptable range. Meanwhile, the corresponding measurement value was taken as prior information. As shown in Figure 6, our marginalization strategy was to decide which frame to remove according to whether the new frame was a keyframe. When the new frame was a keyframe, the oldest keyframe and its corresponding IMU measurements were removed from the sliding window, and the new frame was retained. Otherwise, the image information of the latest keyframe in the sliding window was marginalized, and its corresponding IMU measurements were retained to facilitate subsequent IMU pre-integration. Then, we used the Schur complement [29] to keep important constraint information and ensure calculation consumption.

### 4.6. Loop Closure

**Figure 7:**
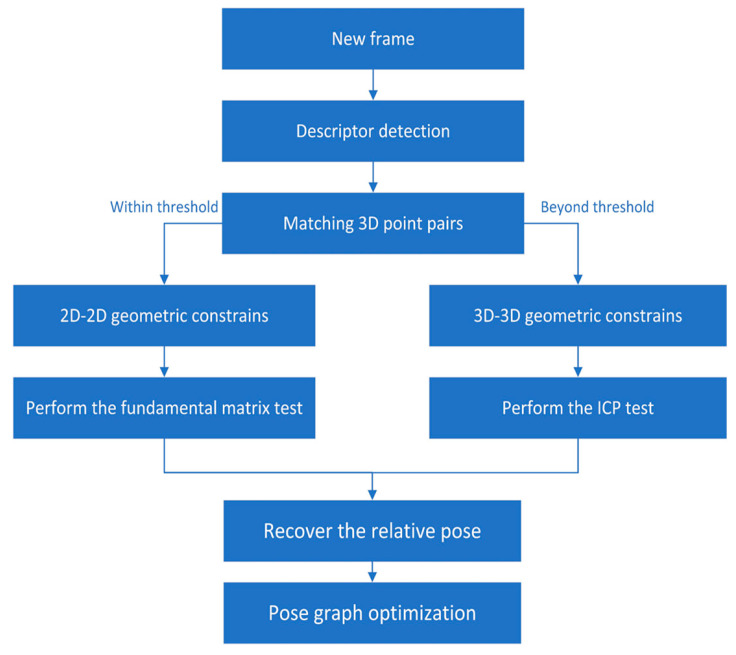
Loop Closure strategy of our system.

The loop closing thread was used to minimize drift accumulated from the backend optimization framework. The detection of loop closures was able to find an image similarity between the new frame and the candidate keyframe followed by estimating a relative pose change between them. We used DboW2 [30] for loop detection, in which only points feature descriptors were used because matching lines across the whole framework were computationally too expensive. When a loop was detected, the connection between the new frame and the candidate keyframe in the local sliding window was established by points feature descriptors. In addition, we used a two-way geometric constraint to compute the relative pose between them. When the 3D points obtained from the new frame were less than the threshold we set, a 2D–2D strategy was used to estimate relative pose. We used 2D observations of points features in the new frame and the candidate keyframe to perform the fundamental matrix test. Otherwise, we performed the ICP test to recover the relative pose. The corresponding strategy is shown in the Figure 7. Finally, a pose graph optimization was launched to the loop closure.

## 5. Experiment

We tested our system on public datasets provided in STAR-Center dataset [31] and the OpenLORIS-Scene dataset [32]. To our knowledge, there is no open source project that proposes an RGB-D-inertial system based on points and lines. We compared our system with four state-of-the-art open source visual systems: PLVIO, based on an optimization method, which uses monocular visual-inertial odometry with points and lines. OKVIS, which is a monocular visual-inertial odometry with point-based modes. VINS-MONO [10] is the state-of-the-art SLAM system: monocular integrated with IMU. LineSLAM, which is an RGB-D odometry based on points and lines and is not integrated with IMU. The open source evaluation tool evo [33] was used to evaluate the absolute pose error (APE), which directly compared the trajectory error between the estimate values of all systems and the ground truth. All of our experiments were conducted on a computer equipped with a i5-8750H CPU at 1.6–2.3 GHz and 8 GB RAM, and no GPU was used.

### 5.1. STAR-Center Dataset

**Figure 8:**
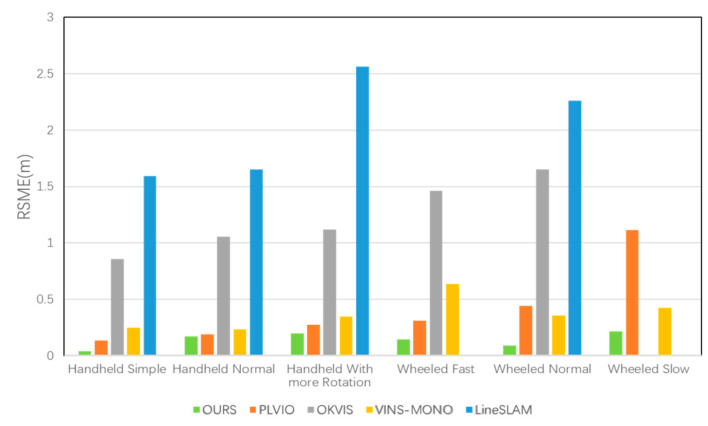
The RSME for OURS, PLVIO, OKVIS, VINS-MONO and LineSLAM in the STAR-Center dataset.

The STAR-Center dataset contained color images and depth images taken by an RGB-D camera at 30FPS and synchronized inertial data at 250 Hz. This dataset included two scenarios: a handheld and a wheeled robot. According to the different rotation degrees, the handheld datasets were divided into three modes: a handheld simple, handheld normal and handheld with more rotation. According to the velocity of the robot, the wheeled robot datasets were also divided into three parts: wheeled slow, wheeled normal, and wheeled fast. The trajectories were gained from different velocities on a road with two up–down slopes. All of these provided the ground truth trajectory captured by the Optitrack tracking system. For quantitative evaluation, we compared our system with four state-of-art SLAM systems without loop closure. Table 1 shows the root mean square error (RMSE) of APE of the trajectory between the estimate and the ground truth, and the corresponding histogram is shown in Figure 8.

**Figure 9:**
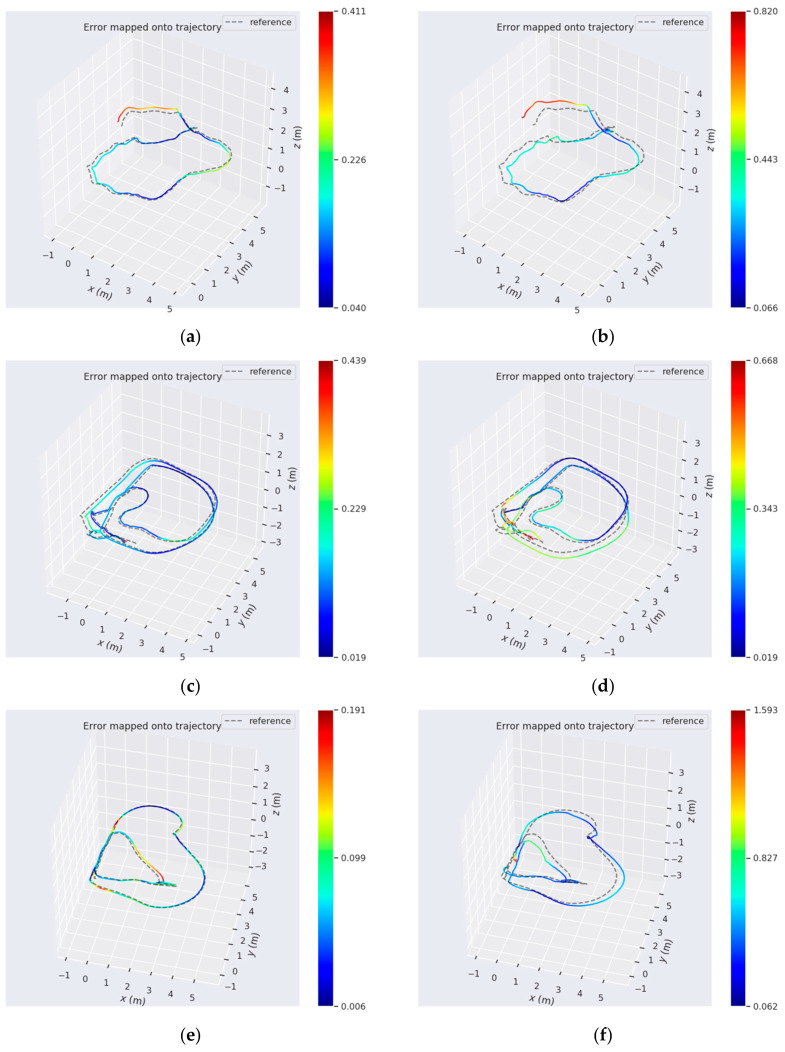
The comparative absolute translation errors in the several datasets. The three colorful trajectories of the left column are run with our system on: (**a**) the Handheld With More Rotation; (**c**) the Wheeled Fast; (**e**) the Wheeled Normal. The trajectories (**b**,**f**) are the results of VINS-MONO on the corresponding data. (**d**) shows the results of PLVIO on the corresponding data.

According to Figure 8, our system, which jointly utilizes points and lines, achieved superior performances for positioning on six sequences. The result in Figure 7 shows that LineSLAM-based RGB-D odometry, which is not integrated with IMU, had the worst performance on all sequences. OKVIS which only relied on point features also performed poorly. PLVIO, based on monocular with points and lines, and VINS-MONO, which is the state-of-art SLAM system, also realized better precision. To further demonstrate the results intuitively, we generated a more specific comparison with PLVIO and VINS-MONO on several sequences: Handheld with More Rotation, Wheeled Fast, and Wheeled Normal. There are several detailed error maps on trajectories estimated by our system, PLVIO and VINS-MONO shown in Figure 9, where the reference represents the ground truth. The more intense the color, the bigger the trajectory error is. Comparing the performances of the three systems, it can be conclued that our algorithm based on RGB-D camera yields smaller errors for all sequences than PLVIO and VINS-MONO based on monocular, especially in the cases of motion with more rotation and fast velocity. The results also demonstrate that our system achieves higher accuracy with changing scenarios and exhibits a better robustness.

**Figure 10:**
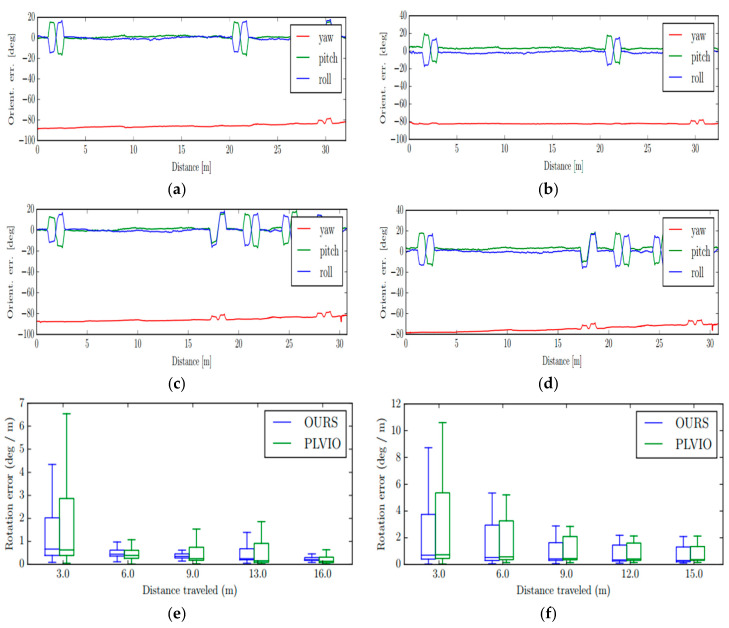
The comparative absolute rotation errors. The first two rows show the absolute orientation error in the Handheld With More Rotation and Wheeled Slow Experiment, respectively, where the left results (**a**,**c**) are from our system and right (**b**,**d**) are from PLVIO. The last row shows the absolute rotation error of our system and PLVIO, where the left results (**e**) are the Handheld with More Rotation Experiment and the right (**f**) is Wheeled Slow Experiment.

Furthermore, in order to make a more comprehensive demonstration state of our system and PLVIO with more rotation and slow velocity, we also compared the rotation errors of the two system under these two scenarios: Handheld With more Rotation and wheeled slow, and the results are displayed in Figure 10. From the rotation error diagrams shown in the Figure 10, it can be seen that our system has better observations of the motion rotation, and performs better in terms of orientation along these trajectories.

### 5.2. OpenLORIS-Scene Dataset

**Figure 11:**
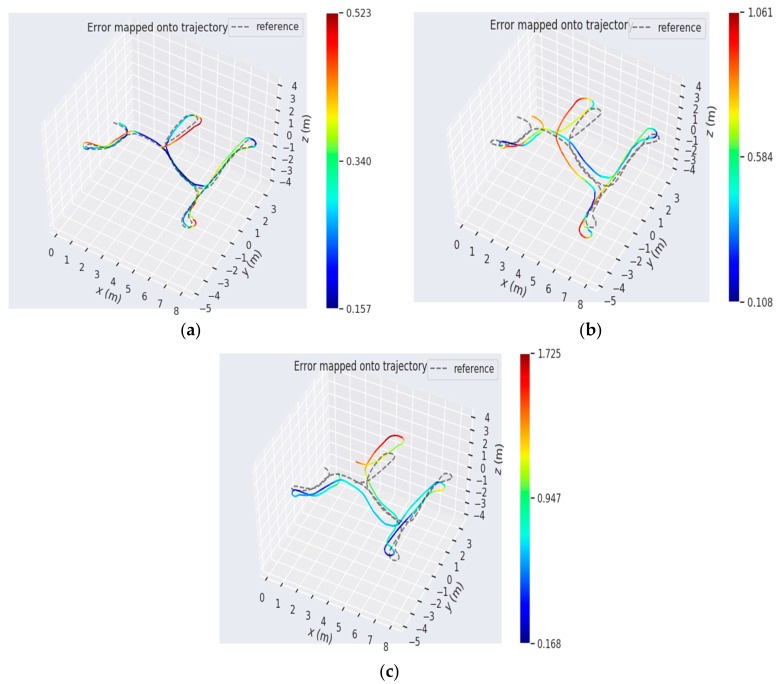
The comparative absolute translation errors. The trajectory heat map estimated by (**a**) our system, (**b**) PLVIO, and (**c**) VINS-MONO in Home1–1.

The OpenLORIS-Scene dataset is the latest SLAM benchmark that recorded the synchronized data of multiple sensor real scenes with a real robot, including: the home, the office, the corridor, the café and the marker scene. All the intrinsic and extrinsic parameters between sensors were well calibrated. The ground truth of trajectory were provided by an OptiTrack MCS and a Hokuyo UTM30LX LiDAR. We used the data collected by the RealSense D435i camera in the home scene, which contains many low textured scenes and motion with rapid rotations. We also compared the proposed system with four system: PLVIO, OKVIS, VINS-MONO and LineSLAM. For fairness, the loop closure module was not used in all systems. Table 2 shows the root mean square error (RMSE) of the translation and rotation of the trajectory between the estimate and the ground truth. It shows that our system provided the best performance on four sequences for the translation, except for Home1–5. In addition, our method performed the best on three sequences for rotation, except for Home1–2 and Home1–4. PLVIO also achieved a highest accuracy on Home1–4 and Home1–5, and VINS-MONO had the best performance at rotation error on the home1–2 dataset. There are several detailed error maps on translation and rotation of trajectories estimated by our system, PLVIO and VINS-MONO, as shown in Figure 11.

From Table 2 and Figure 11, it can be seen that our system has the higher accuracy on pose estimation in a home low texture scene. Compared with the SLAM system which only relies on point feature or is based on monocular, the proposed system gives a smaller error on translation and rotation of motion and achieves a better robustness.

### 5.3. Running Time Performance Evaluation

In this section, we first evaluate the initialization process of our system and compare the average time of the initialization with PLVIO and VINS-MONO on the STAR-Center dataset and the OpenLORIS-Scene dataset. Table 3 shows the execution time of each dataset. We can observe that PLVIO and VINS-MONO takes a longer time to conduct visual SFM and align visual and IMU measurements during initialization for all eleven datasets. The result shows that our system achieved a faster initialization process.

In order to further demonstrate the real-time improvement of the system, we evaluated the running time of the whole backend the system specifically. We compared it with PL-VIO in all sequences, which is also a VIO system with points and lines. Both of their backend process could be divided into the initialization process and the optimization process. Experiments on each of the sequences measured the running time of the backend process in a sliding window. The results are shown in Table 4. It can be seen from Table 4 that due to the rapidity in the initialization process of our system, the optimization process was also accelerated. We achieved a more real-time backend process and improved the operation efficiency of the whole system.

## 6. Conclusions

In this work, a novel tight-coupled RGB-D inertial SLAM with multiple features is proposed, which fuses color images, depth images, and IMU information for motion estimation. Our system contains two main modules: the frontend and the backend. In the frontend, points and lines are detected and matched; IMU states are also propagated by IMU pre-integration. The keyframes are selected by the number of matched features and IMU measurements.

In the backend, first, a simplified global initialization is achieved by directly obtaining 3D landmarks for every frame, resulting in the system having better initial pose estimation. A line feature represented in Plücker coordinates is used for triangulation, and its orthonormal representation is employed to optimize the system state. A factor graph is applied to show the relationships in our system state. In addition, we present a novel nonlinear optimization framework to minimize the residuals of visual features and IMU measurements in sliding windows. Moreover, the Jacobian matrices of the cost function are derived in an effective way, which is crucial for solving the optimization problem. Finally, a comparison with four other state-of-the-art open-source systems on public datasets is provided. The experimental results on two datasets show that our system can achieve the highest pose estimation and robustness of all cases. Meanwhile, specific experiments are carried out, and the results also demonstrate the better performance of our system in terms of rotation error and translation. The experiment regarding initialization confirms that our system achieves a faster initialization than the monocular system, which ensures a good real-time performance of the system. Moreover, we achieved a more real-time backend process and improved the operation efficiency of the whole system. In the future, we plan to find more ways to set up the constraints of 3D line segments, and a real-time dense 3D map will be realized to develop an entire navigation system.

## Figures and Tables

**Table 1:** The root mean square error (RMSE) of translation on STAR-Center dataset in meters. “-” denotes test failed.

Sequences	OURS	PLVIO	OKVIS	VINS-MONO	LineSLAM
Handheld Simple	**0.0390**	0.1351	0.8568	0.2457	1.5920
Handheld Normal	**0.1728**	0.1885	1.0563	0.2355	1.6528
Handheld With more Rotation	**0.1966**	0.2768	1.1203	0.3486	2.5645
Wheeled Fast	**0.1433**	0.3096	1.4609	0.6355	-
Wheeled Normal	**0.0888**	0.4411	1.6508	0.3548	2.2615
Wheeled Slow	**0.2169**	1.1127	-	0.4252	-

The numbers in bold represent the estimated trajectory is more close to the ground truth trajectory.

**Table 2:** The root mean square error (RMSE) of translation and rotation on the home scene dataset in meters.

Seq.	OURS		PLVIO		OKVIS		VINS-MONO		LineSLAM	
	Trans.	Rot.	Trans.	Rot.	Trans.	Rot.	Trans.	Rot.	Trans.	Rot.
Home1–1	**0.3459**	**0.1732**	0.5427	0.1787	0.9085	0.2056	0.7374	0.2974	1.2452	0.4589
Home1–2	**0.3178**	0.4209	0.3239	0.5762	0.7752	0.5641	0.7056	**0.3445**	1.3598	0.6841
Home1–3	**0.3391**	**0.1481**	0.3780	0.1597	0.5618	0.3541	0.5154	0.1554	1.4526	0.6485
Home1–4	**0.3174**	0.1727	0.5742	**0.1702**	0.8415	0.2946	0.3545	0.1771	1.5256	0.7895
Home1–5	0.2366	**0.1192**	**0.2324**	0.1205	0.6845	0.2649	0.2551	0.3616	0.9212	0.5684

The numbers in bold represent the estimated trajectory is closer to the ground truth trajectory.

**Table 3:** Execution time of the initialization in milliseconds.

Sequences	OURS	PL-VIO	VINS-MONO
Handheld Simple	**37.2737**	149.068	110.365
Handheld Normal	**38.621**	139.913	98.465
Handheld With More Rotation	**16.9961**	107.579	80.461
Wheeled Fast	**58.937**	170.996	94.12
Wheeled Normal	**80.6177**	155.938	124.25
Wheeled Slow	**78.2723**	205.115	151.32
Home1–1	**45.62**	126.38	125.36
Home1–2	**78.45**	134.65	114.57
Home1–3	**90.54**	148.63	120.24
Home1–4	**34.56**	107.38	70.65
Home1–5	**60.17**	124.36	70.54

The numbers in bold represent the faster running time.

**Table 4:** Execution time of the whole backend in milliseconds.

Sequences	OURS			PL-VIO		
	Initialization	Optimization	Backend	Initialization	Optimization	Backend
Handheld Simple	**37.273**	**57.254**	**94.527**	149.068	106.245	255.313
Handheld Normal	**38.621**	**49.245**	**87.866**	139.913	84.451	224.364
Handheld With More Rotation	**16.996**	84.254	**101.25**	107.579	**60.245**	167.824
Wheeled Fast	**58.937**	**78.245**	**137.182**	170.996	106.245	277.241
Wheeled Normal	**80.617**	120.453	**201.07**	155.938	**94.156**	250.094
Wheeled Slow	**78.272**	**113.245**	**191.517**	205.115	163.145	368.26
Home1–1	**45.62**	**60.254**	**105.874**	126.38	65.215	191.595
Home1–2	**78.45**	**90.214**	**168.664**	134.65	105.364	240.014
Home1–3	**90.54**	**36.854**	**127.394**	148.63	70.548	219.178
Home1–4	**34.56**	**60.866**	**97.426**	107.38	101.593	208.973
Home1–5	**60.17**	**70.214**	**130.384**	124.36	120.648	245.008

The numbers in bold represent the faster running time.
